# Interaction of Cx43 with Hsc70 regulates G1/S transition through CDK inhibitor p27

**DOI:** 10.1038/srep15365

**Published:** 2015-10-20

**Authors:** Hitoshi Hino, Ping Dai, Tatsushi Yoshida, Tomoya Hatakeyama, Yoshinori Harada, Eigo Otsuji, Tsukasa Okuda, Tetsuro Takamatsu

**Affiliations:** 1 Department of Pathology and Cell Regulation, Kyoto Prefectural University of Medicine, Kyoto, Japan; 2 Division of Digestive Surgery, Department of Surgery, Kyoto Prefectural University of Medicine, Kyoto, Japan; 3 Department of Cellular Regenerative Medicine, Kyoto Prefectural University of Medicine, Kyoto, Japan; 4 Department of Biochemistry and Molecular Biology, Kyoto Prefectural University of Medicine, Kyoto, Japan

## Abstract

Connexin 43 (Cx43) functions as a cell growth suppressor. We have demonstrated that Cx43 interacts with heat shock cognate protein 70 (Hsc70) for regulating cell proliferation. Hsc70 interacts with CDK inhibitor p27, which regulates the assembly and subcellular localization of cyclin D1-CDK4-p27 complex. However, the involvement of p27 with Cx43-mediated cell cycle suppression is still poorly understood. Here, we report that nuclear accumulation of p27 is reduced by overexpression of Cx43, and that this reduction is restored by co-overexpression with Hsc70. We found that Cx43 competes with p27 for binding to Hsc70, and as a result, decreases the level of Hsc70 in cyclin D1-CDK4-p27 complex, leading to prevention of the nuclear translocation of the complex and the G1/S transition. Collectively, our findings suggest that, in Cx43 up-regulation, which is most likely an emergency measure, Cx43-Hsc70 interaction regulates cell cycle G1/S progression through a novel mechanism by which Cx43-Hsc70 interaction prevents the nuclear accumulation of p27 through controlling the nuclear translocation of cyclin D1-CDK4-p27 complex.

Connexins, a family of transmembrane proteins, form gap junction channels within the membranes between contacting cells^1^,^2^. Gap junctional intercellular communication (GJIC) is considered to have crucial roles in regulating normal cell growth, differentiation, development and homeostasis^1^,^2^,^3^. Connexin43 (Cx43) is one of the most frequently investigated connexin proteins and among the most widely expressed connexin genes in human tissues^4^. Cx43 has long been considered cell growth suppressor through providing cell-cell communication via GJIC^5^,^6^,^7^. More recently, it was also documented that Cx43 controls cell growth through mechanisms independent of GJIC^8^,^9^,^10^,^11^,^12^,^13^, e.g., protein-protein interaction. Cx43 has a unique long carboxy-terminal (CT) tail. This CT domain is reported to be the binding site of multiple proteins, and these protein-protein interactions are involved in the regulation of physiological events^14^.

To elucidate the molecular mechanisms of Cx43-mediated cell cycle suppression, we have determined that heat shock cognate protein 70 (Hsc70) functions as a novel Cx43-interacting protein^15^. Hsc70 is expressed ubiquitously and constitutively, regardless of cell stress, and is involved in cell cycle progression^16^. We demonstrated that Cx43 competes with cyclin D1, which functions as a positive regulator of cell cycle mainly in the nucleus, for binding to Hsc70^15^,^17^. Moreover, overexpression of Cx43 decreases the nuclear accumulation of cyclin D1 in a GJIC-independent manner, and as a result, the G1/S transition is negatively regulated.

The progression of the cell cycle is regulated by the activities of various cyclin-dependent kinases (CDKs). In addition, these are negatively regulated by a group of proteins, cell cycle regulators, collectively termed CDK inhibitors (CKIs). Regarding the association between CKIs and Cx43, it has already been reported that Cx43 increases the expression level of p27, a member of the Cip/Kip family, and inhibits cell proliferation^13^. However, unexpectedly, we found that nuclear accumulation of p27 is reduced by overexpression of Cx43^15^. This subcellular movement of p27 is seemingly contradictory, since the nuclear accumulation of p27 is considered to function negatively during the cell cycle^18^,^19^,^20^. On the other hand, previous reports showed that Hsc70 directly interacts with p27^21^,^22^,^23^. Cytoplasmic cyclin D1, CDK4 and p27 form a complex^24^,^25^,^26^,^27^, and this assembled complex is imported to the nucleus in the mid- to late G1 phase^18^. Moreover, Hsc70 is also included in the complex of cyclin D1-CDK4-p27^28^. Overexpression of Cx43 decreased the nuclear accumulation of both cyclin D1 and p27^15^. These findings led us to speculate that Cx43 would regulate the cyclin D1-CDK4-p27 complex via interaction with Hsc70, and as a result, subcellular distribution of p27 would be regulated in Cx43-Hsc70 interaction–mediated cell cycle suppression.

To elucidate this unexpected molecular relationship between Cx43 and p27, we examined whether reduced nuclear accumulation of p27 by overexpressed Cx43 is due to the prevention of the nuclear translocation of cyclin D1-CDK4-p27 complex by Cx43-Hsc70 interaction, and consequently, whether G1/S transition is inhibited by Cx43. Our findings suggest that Cx43-Hsc70 interaction-mediated p27 subcellular distribution could be a novel mechanism in Cx43 regulating cell cycle progression.

## Results

### Upregulation of Cx43 suppresses the cell proliferation via affecting the nuclear accumulation of p27

**Figure 1:**
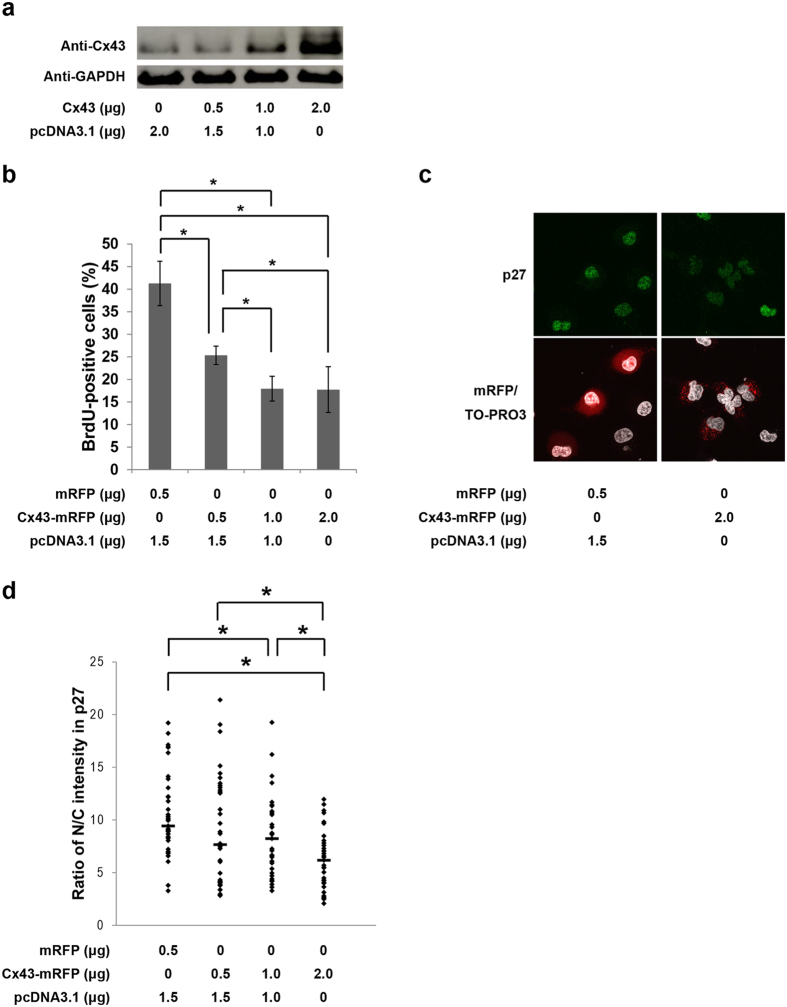
Effects of Cx43 expression on G1/S transition and subcellular localization of p27. (**a**) Expression level of the exogeneouse Cx43 was confirmed by Western blotting with anti-Cx43. GAPDH was used as a loading control. (**b**) BrdU incorporation assay in HuH-7 cells. HuH-7 cells were transfected with mRFP as an indicator and control empty vector, or the dose of Cx43-mRFP indicated in the figure. After 48 hr of transfection, at least 100 transfected cells were counted in each sample, and the percentages of BrdU-positive cells were determined. Data are presented as mean and SD (n = 4, *p < 0.05). (**c**) Effect of Cx43 on subcellular localization of p27. HuH-7 cells were transfected with mRFP or Cx43-mRFP. After 48 hr of transfection, immunofluorescence stainings were performed with anti-p27 antibody. (**d**) Ratio of nuclear/cytoplasmic (N/C) fluorescence intensities in p27. HuH-7 cells were transfected with mRFP or the indicated dose of Cx43-mRFP. After 48 hr of transfection, immunofluorescence staining was carried out with anti-p27 antibody and nucleocytoplasmic localization analysis in p27 was performed. At least 30 transfected cells were examined and quantified for each group, and the data were plotted. The horizontal lines represent median values. *p < 0.05.

To study in more detail whether upregulation of Cx43 also inhibits the cell proliferation of HuH-7 cells, which are derived from human hepatocellular carcinoma cells (HCCs) and express endogenous Cx43, HuH-7 cells were transfected with increased level of Cx43-expressing vector, Cx43-mRFP. The percentages of transfected cells in S phase in each group were evaluated using BrdU incorporation assays. After 48 hr of transfection, in the empty vector mRFP-transfected group, 41% BrdU-positive cells were observed. However, a marked decrease in S phase was observed, by 25%, 18% and 18% in 0.5 μg, 1.0 μg and 2.0 μg of Cx43-mRFP transfected cells, respectively (Fig. 1b).

Next, to investigate whether the Cx43-mediated cell cycle suppression is due to a correlation between the expression level of Cx43 and nuclear accumulation of p27, HuH-7 cells were transfected with either empty vector mRFP or Cx43-mRFP. The nuclear accumulation of p27 was clearly decreased in Cx43-mRFP transfected cells compared with mRFP transfected cells at 48 hr after transfection (Fig. 1c). The quantitative analysis showed that the ratio of nuclear intensity of p27 was decreased by Cx43 in a dose-dependent manner (Fig. 1d).

### Upregulation of Hsc70 restores Cx43-reduced nuclear accumulation of p27

**Figure 2:**
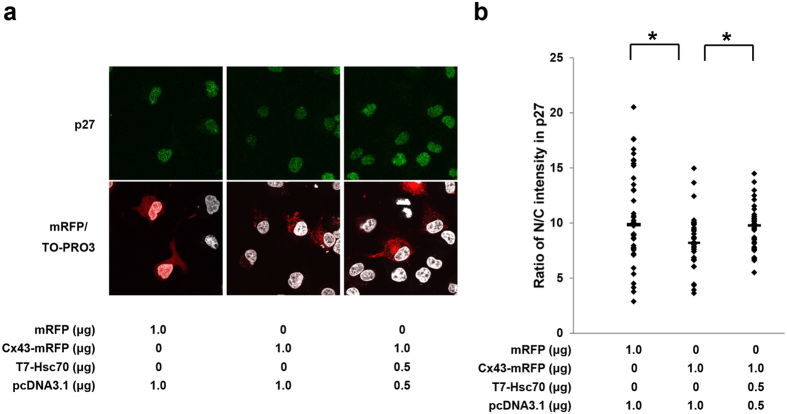
Cx43-Hsc70 interaction decreases nuclear accumulation of p27. (**a**) Effect of Cx43-Hsc70 interaction on nuclear accumulation of p27. HuH-7 cells were transfected with Cx43-mRFP with or without T7-Hsc70. After 48 hr of transfection, immunofluorescence staining with anti-p27 antibodies was carried out. (**b**) Ratio of nuclear/cytoplasmic (N/C) fluorescence intensities in p27. Nucleocytoplasmic localization analysis in p27 was performed. At least 30 transfected cells were examined and quantified for each group, and the data were plotted. The horizontal lines represent median values. *p < 0.05.

The Cx43-mediated cell cycle suppression can be restored by co-overexpression with Hsc70 in HuH-7 cells^15^. To determine whether Cx43 overexpression-decreased nuclear accumulation of p27 can be restored by co-overexpression with Hsc70, HuH-7 cells were transfected with Cx43-mRFP alone or with both Cx43-mRFP and T7-Hsc70. As expected, co-overexpression of Cx43 with Hsc70 restored the nuclear accumulation of p27 in HuH-7 cells (Fig. 2a). Moreover, the quantitative analysis showed that the Cx43-reduced ratio of nuclear to cytoplasmic intensity of p27 was abolished by co-overexpression with Hsc70 (Fig. 2b). These experimental data demonstrated that the interaction of Cx43-Hsc70 regulates the nuclear accumulation of p27 as well as cyclin D1. Similar results were also obtained in PANC-1 cells, which are derived from human pancreatic cancer cells and express endogenous Cx43^29^ (see Supplementary Fig. S1).

### Cx43 competitively inhibits the binding of p27 to Hsc70

**Figure 3:**
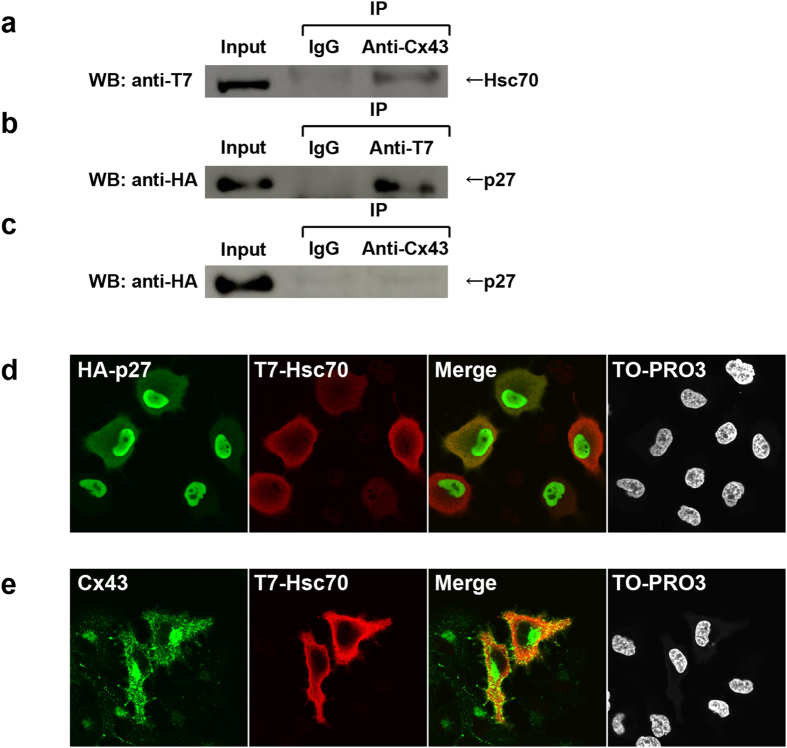
Association among Cx43, Hsc70 and p27. (**a**) Hsc70 interacted with Cx43. Lys**a**tes prepared from HuH-7 cells transfected with T7-Hsc70 and wild-type Cx43 were immunoprecipitated by anti-Cx43 antibody or control IgG. The immunocomplexes were analyzed on 12% SDS-PAGE followed by western blotting (WB) using anti-T7 antibody. (**b**) Hsc70 interacted with p27. Lysates prepared from HuH-7 cells transfected with T7-Hsc70 and HA-p27 were immunoprecipitated by anti-T7-tag antibody or control IgG. The immunocomplexes were analyzed on 12% SDS-PAGE followed by WB using anti-HA-tag antibody. (**c**) Absence of interaction between Cx43 and p27. Lysates prepared from HuH-7 cells transfected with both wild-type Cx43 and HA-p27 expression plasmids were immunoprecipitated by anti-Cx43 antibody or control IgG. The immunocomplexes were analyzed on 12% SDS-PAGE followed by WB using anti-HA antibody. (**a**–**c**) Cropped blots were shown, and full-length blots are presented in Supplementary Fig. S2a, S2b and S2c, respectively. (**d**) Partial subcellular co-localization of p27 and Hsc70. HuH-7 cells were transfected with HA-p27 and T7-Hsc70. Cells were fixed and stained with anti-HA-tag and anti-T7-tag antibodies. (**e**) Partial subcellular co-localization of Cx43 and Hsc70. HuH-7 cells were transfected with Cx43 and T7-Hsc70. Cells were fixed and stained with anti-Cx43 and anti-T7-tag antibodies.

Next, we asked whether Hsc70 interacts with p27 or Cx43. Co-immunoprecipitation assays were performed. HuH-7 cells were co-transfected with T7-Hsc70 with Cx43 (Fig. 3a) or with HA-p27 (Fig. 3b), and with HA-p27 with Cx43 (Fig. 3c). Hsc70 was co-immunoprecipitated with Cx43 (Fig. 3a) and/or with p27 (Fig. 3b). However, no interaction between Cx43 and p27 was detected (Fig. 3c). Furthermore, to illuminate whether there is a subcellular interaction between Hsc70 and p27, or Cx43, we analyzed the distribution of Hsc70, p27 and Cx43 by fluorescence immunostainings with corresponding antibodies. In HuH-7 cells, partial co-localization of Hsc70 with p27 (Fig. 3d) or Cx43 (Fig. 3e) was observed in the cytoplasm.

**Figure 4:**
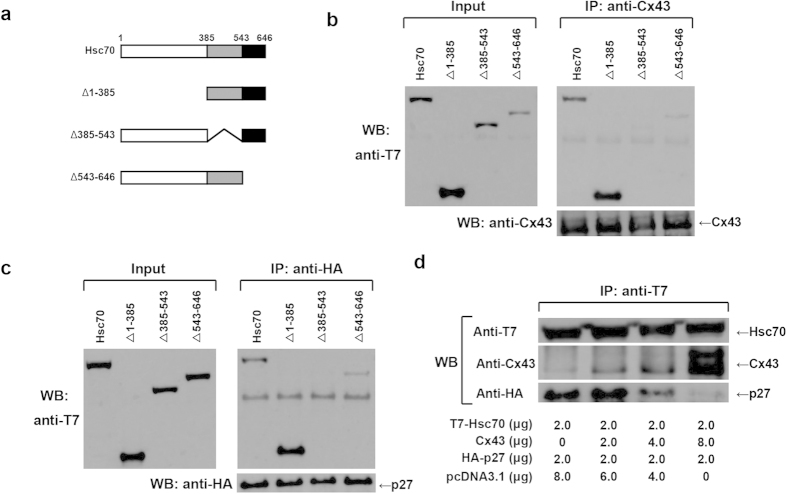
Cx43 competes with p27 for binding to Hsc70. (**a**) A schematic representation of Hsc70 and its deletion mutants is shown. (**b**,**c**) Identification of Cx43 (b) or p27 (**c**) binding domain in Hsc70. HuH-7 cells were transfected with expression vectors encoding Cx43 or HA-p27 and various forms of Hsc70 used in co-immunoprecipitation assays. The immunocomplexes were analyzed on 12% SDS-PAGE followed by western blotting (WB) using anti-T7-tag and anti-Cx43 or anti-HA-tag antibodies. Cropped blots are shown. (**d**) Cx43 and p27 competitively bound to Hsc70. HuH-7 cells were transfected with equivalent plasmids of T7-Hsc70 and HA-p27, and with increasing amounts of Cx43 as indicated in the figure. Lysates were immunoprecipitated by anti-T7 antibody. The immunocomplexes were analyzed on 12% SDS-PAGE followed by WB using anti-T7-tag, anti-Cx43 and anti-HA-tag antibodies. Cropped blots are shown, and full-length blots are presented in Supplementary Fig. S2d.

To investigate the binding association among Cx43, p27 and Hsc70, we determined whether both Cx43 and p27 bind to the same region in Hsc70. A series of T7-tagged Hsc70 expression vectors encoding various portions of the Hsc70 molecule^21^ were transfected into HuH-7 cells (Fig. 4a). Co-immunoprecipitation assays were carried out using the whole lysates extracted from HuH-7 cells. As shown as Fig. 4b, Cx43 bound to wild-type Hsc70, but not to Δ385–543 mutant, which lacks the protein-binding domain in Hsc70 (Fig. 4b, right panel). Δ1–385 and Δ543–646 mutant Hsc70 did not affect binding to Cx43. In addition, similar interactions between Hsc70 and p27 were also observed by co-immunoprecipitation assay (Fig. 4c, right panel). Thus, the same region containing amino acids 385–543 of Hsc70 is required for binding to Cx43 or p27.

Secondly, based on these results, we then reasoned that Cx43 could compete with p27 for interaction with Hsc70. We performed competition assays. HuH-7 cells were transfected with equivalent plasmids of Hsc70, p27 and increasing amount of Cx43 expression plasmids for competitive binding to Hsc70. As a result, Cx43 interfered with the binding of p27 to Hsc70 in a dose-dependent manner (Fig. 4d).

### Competitive binding of Cx43 with p27 to Hsc70 regulates the nuclear accumulation of cyclin D1-CDK4-p27 complex

**Figure 5:**
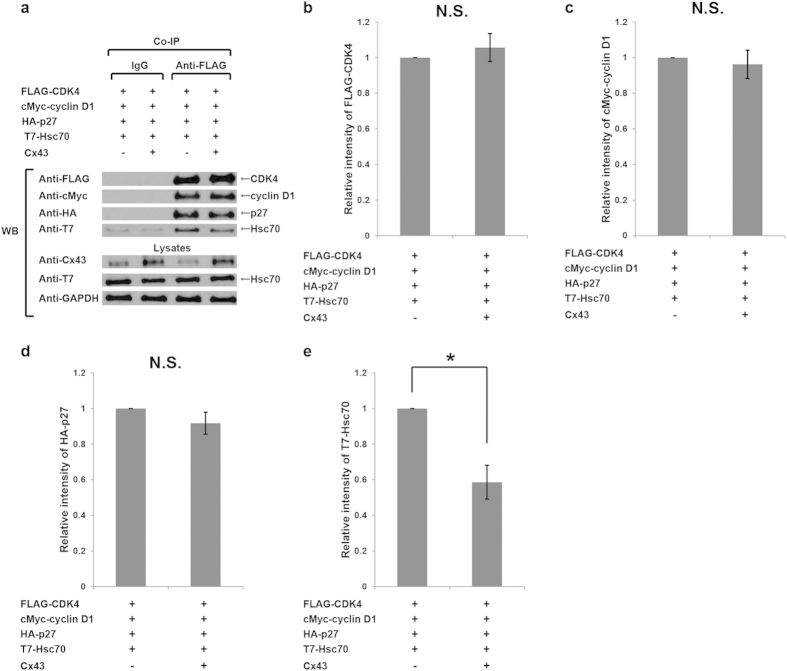
Upregulation of Cx43 decreases the amount of Hsc70 in cyclin D1-CDK4-p27 complex. (**a**) Effects of Cx43 expression on Hsc70 in cyclin D1-CDK4-p27 complex and on components of the complex. HuH-7 cells were transfected with equivalent plasmids of HA-p27, cMyc-cyclin D1, FLAG-CDK4 and T7-Hsc70 with or without Cx43. Lysates were immunoprecipitated by control IgG or anti-FLAG-tag antibody. The immunocomplexes were analyzed on 12% SDS-PAGE followed by western blotting (WB) using anti-FLAG-tag, anti-HA-tag, anti-cMyc-tag and anti-T7 tag antibodies. Cropped blots are shown. (**b**–**e**) Relative intensity levels of FLAG-CDK4 (**b**), cMyc-cyclin D1 (**c**), HA-p27 (**d**) and T7-Hsc70 (**e**) were quantified and normalized in Cx43-overexpressed groups against those of non-Cx43-overexpressed groups, respectively (n = 3, *p < 0.05).

Cell cycle progression, particularly the G1/S transition, is negatively controlled by CKIs. CKI protein p27 regulates the cyclin D1-CDK4 complex by sequestration^30^. The Cx43-Hsc70 interaction results in prevention of the nuclear accumulation of cyclin D1 during G1/S transition^15^. To elucidate whether Cx43-Hsc70 interaction negatively regulates the nuclear accumulation of p27 through preventing the nuclear translocation of cyclin D1-CDK4-p27 complex, HuH-7 cells were co-transfected with FLAG-CDK4, cMyc-cyclin D1, HA-p27 and T7-Hsc70 with or without Cx43. Co-immunoprecipitation assays were performed using the whole lysates extracted from HuH-7 cells. No changes in the amounts of CDK4 (Fig. 5a,b), cyclin D1 (Fig. 5a,c), and p27 (Fig. 5a,d) in cyclin D1-CDK4-p27 complex were observed. However, the amount of Hsc70 in the complex was remarkably reduced by co-overexpression with Cx43 (Fig. 5a,e). Since total amount of Hsc70 in the whole lysates was not altered, these results indicate that Cx43 inhibits the interaction of CDK4 and Hsc70 without any change of Hsc70 expression nor degradation.

**Figure 6:**
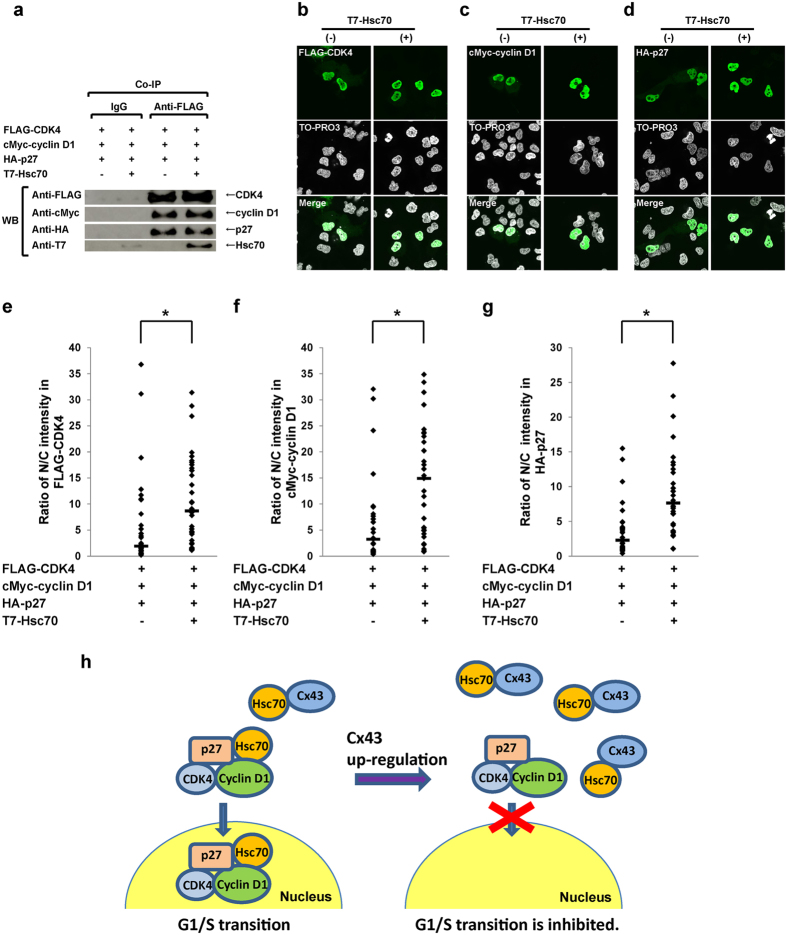
Hsc70 regulates nuclear translocation of cyclin D1-CDK4-p27 complex. (**a**) Hsc70 did not affect the assembly of cyclin D1-CDK4-p27 complex. HuH-7 cells were transfected with equivalent plasmids of HA-p27, cMyc-cyclin D1and FLAG-CDK4 with or without T7-Hsc70. Lysates were immunoprecipitated by control IgG or anti-FLAG-tag antibody. The immunocomplexes were analyzed on 12% SDS-PAGE followed by western blotting (WB) using anti-FLAG-tag or anti-HA-tag, and/or anti-cMyc-tag, or anti-T7 tag antibodies. Cropped blots are shown. (**b**–**d**) Subcellular localization of the complex components CDK4 (**b**), cyclin D1 (**c**) and p27 (**d**). HuH-7 cells were transfected equivalent plasmids of HA-p27, cMyc-cyclin D1 and FLAG-CDK4 with or without T7-Hsc70. After 48 hr of transfection, immunofluorescence staining with anti-FLAG-tag or anti-cMyc-tag, and/or anti-HA-tag antibodies was carried out, respectively. (**e**–**g**) Ratio of nuclear/cytoplasmic (N/C) fluor**e**scence intensities in CDK4 (**e**), cyclin D1 (f), and p27 (**g**). Nucleocytoplasmic localization analysis was performed in FLAG-tag, cMyc-tag and HA-tag, respectively. At least 30 transfected HuH-7 cells were examined and quantified for each sample. The data were plotted, and the horizontal lines represent median values. *p < 0.05. (**h**) A schematic illustration of the mechanism by which Cx43 regulates G1/S transition. Hcs70 interacts with the cyclin D1-CDK4-p27 complex and enhances the nuclear translocation of the complex, leading to G1/S transition (left). When Cx43 is up-regulated (right), the Cx43-Hsc70 interaction prevents the cyclin D1-CDK4-p27 complex enhancement for the nuclear translocation, causing inhibition of G1/S transition.

Next, to demonstrate whether the change in the amount of Hsc70 in cyclin D1-CDK4-p27 complex affects the subcellular distribution of the complex components of cyclin D1, CDK4 and p27, HuH-7 cells were co-transfected with cMyc-cyclin D1, HA-p27 and FLAG-CDK4 with or without T7-Hsc70. In co-immunoprecipitation assays, in spite of the presence or absence of Hsc70 in the complex, no changes of amounts of the complex components of CDK4, cyclin D1 and p27 were observed (Fig. 6a). However, compared with non-co-transfected with Hsc70 cells, significant increase of nuclear accumulation of the complex components of CDK4 (Fig. 6b,e), cyclin D1 (Fig. 6c,f), and p27 (Fig. 6d,g) were observed in those co-transfected with Hsc70 cells.

Furthermore, we knocked down endogenous Hsc70 in HuH-7 cells by co-transfected FLAG-CDK4, cMyc-cyclin D1 and HA-p27 with or without siRNA of Hsc70. Endogenous Hsc70 was efficiently reduced by transfection with the siRNA (see Supplementary Fig. S3a). As expected, the amounts of CDK4, cyclin D1 and p27 in the complex were not affected by knockdown of Hsc70 (see Supplementary Fig. S3b). However, the nuclear accumulations of CDK4 (see Supplementary Fig. S3c), cyclin D1 (see Supplementary Fig. S3d) and p27 (see Supplementary Fig. S3e) were suppressed in Hsc70-knockdown cells.

## Discussion

Cx43 has been considered a cell cycle suppressor^5^,^6^,^7^,^8^,^9^,^10^,^11^,^12^,^13^. However, how Cx43 mediates cell growth is not fully elucidated. In this study, first, we demonstrated that Cx43 suppresses G1/S transition of HuH-7 cells (Fig. 1) and PANC-1 cells (see Supplementary Fig. S1) though it induces a reduction in the level of nuclear accumulation of p27 in a dose-dependent manner. Second, Cx43 competed with p27 for binding to Hsc70 (Fig. 4), and overexpression of Hsc70 could restore the reduced nuclear accumulation of p27 by overexpression of Cx43 (Fig. 2 and see Supplementary Fig. S1). In addition, the Cx43-mediated cell cycle suppression can be restored by co-overexpression with Hsc70 in HuH-7 cells^15^ and PANC-1 cells (see Supplementary Fig. S1). Third, overexpression of Cx43 reduced the amount of Hsc70 in the cyclin D1-CDK4-p27 complex (Fig. 5), and the level of Hsc70 in the complex contributed to the nuclear translocation of this complex (Fig. 6). Collectively, these results suggest that Cx43 suppresses cell cycle progression probably by preventing the nuclear accumulation of p27 via the interaction with Hsc70. Moreover, the amount of Hsc70 in cyclin D1-CDK4-p27 complex was regulated by Cx43 via competitive binding with p27 to Hsc70, and consequently, Cx43 controlled the nuclear translocation of cyclin D1-CDK4-p27 complex. Thus, our findings suggest that a novel mechanism of Cx43 suppression of cell growth occurs by negatively regulating the nuclear accumulation of cyclin D1-CDK4-p27 complex through interaction with Hsc70.

The amount of p27 is large during the quiescent G0 phase of the cell cycle, while it is rapidly decreased on reentry of the cells from the G0 into the G1 phase^18^,^20^. Nuclear p27 is considered to function negatively during the cell cycle^19^,^20^. In proliferating cells, p27 is degraded in the nucleus during S and G2 phases by S-phase kinase-associated protein 2 (Skp2), the F-box protein component of the SCF ubiquitin ligase (E3) complex^31^. However, in our previous experiments, overexpression of Cx43 was not found to increase the expression level of p27, although knockdown of Skp2 increased the expression level of nuclear p27 in HuH-7 cells^15^. In fact, a number of clinical studies showed that decreased expression of p27 is closely associated with malignant potential and/or poor prognoses in various types of human cancers^18^,^32^. However, it was also reported that the expression level of p27 is significantly increased in some highly proliferative cancer cells^33^,^34^,^35^, suggesting that p27 does not simply act as a negative regulator of the cell cycle. Moreover, in p27-overexpressing human HCCs with increased cell proliferation, the function of p27 as a negative regulator of cell cycle is inactivated, and this inactivation is closely associated with the sequestration of p27 into cyclin D1-CDK4-containing complexes^35^,^36^. Based on these lines of evidence, it is speculated that formation of the cyclin D1-CDK4-p27 complex would be more crucial for regulating cell cycle progression than nuclear expression of p27 under some particular conditions. In addition, as for hepatocytes, Cx43 is upregulated in the disease conditions such as inflammation and hepatic carcinogenesis^37^,^38^,^39^, and increased Cx43 expression is considered to be an adaptive protective response of the liver^38^. Therefore, we speculate that up-regulated Cx43 functions as a negative regulator of the cell cycle by controlling the cyclin D1-CDK4-p27 complex in p27-overexpressing HCCs and/or other cancers with high proliferative activity.

On the other hand, Hsc70 is well known as a molecular chaperon involved in the progression of cell cycle, regardless of cell stress^21^,^22^,^23^,^28^,^40^, and contributes to the nuclear transport of some proteins, including cyclin D1^23^,^41^,^42^,^43^. In our studies, the amounts of CDK4, cyclin D1 and p27 in the complex were not affected by up- or down-regulating the level of Hsc70 (Fig. 6a and See Supplementary Fig. S3b). However, the subcellular distribution, i.e., the nuclear accumulation of cyclin D1, CDK4 and p27, was influenced by the level of Hsc70 (Fig. 6b–d). This evidence suggests that Hsc70 functions as a chaperon for the complex, and raises the interesting possibility that Cx43 competes with Hsc70-associated cyclin D1-CDK4-p27 complex in the cytoplasm through directly binding to Hsc70, and as a result, the amount of Hsc70 for nuclear translocation of the complex is reduced, and the accumulation of p27 in the nucleus is prevented, by Cx43. Therefore, the mechanism by which Cx43 suppresses the cell cycle progression in our experimental cell lines seems to be independent of the Skp2-regulated p27-degradation pathway. In addition, the reduced nuclear accumulation of p27 would be correlated with the cell cycle suppression in some cell lines only in cases in which nuclear translocation of cyclin D1-CDK4-p27 complex was prevented.

In Cx43-negative cell line, U2O2 cells, overexpressed Cx43 suppresses the cell cycle by increasing the expression level of p27, and decreases the expression level of Skp2^13^. Our present data seem to conflict with the research reports on p27 so far. Previous reports showed that p27 promotes the assembly of cyclin D1-CDK4 or CDK6-p27 complexes and stabilizes them in cytoplasm^24^,^25^,^26^,^27^. Cells deficient in p27 do not assemble the cyclin D-CDK complexes. Thus, p27 is essential for activation of CDKs^26^. Moreover, p27 could facilitate nuclear import of cyclin D1-CDK4 or CDK6-p27 complexes to contribute to G1/S transition^24^. Based on this evidence and our findings, we reason that Cx43 inhibits the chaperon function of Hsc70 for nuclear import of cyclin D1-CDK4-p27 complex via sequestrating Hsc70 in the cytoplasm, and as a result, cell cycle progression is suppressed by Cx43.

In summary, we demonstrated that Cx43 functions as a cell cycle suppressor in HuH-7 and PANC-1 cells through a p27 degradation-independent pathway. Our data provide the first evidence that the interaction of Cx43 with Hsc70 prevents Hsc70-promoting nuclear translocation of cyclin D1-CDK4-p27 complex, resulting in reduction of nuclear accumulation of p27. Collectively, our current findings suggest that Cx43-Hsc70 interaction is involved in suppressing the G1/S cell cycle progression through a novel mechanism by which Cx43 reduces nuclear accumulation of p27 by inhibiting the chaperon function of Hsc70 for nuclear import of cyclin D1-CDK4-p27 complex.

## Methods

### Plasmids

Various forms of pCAT7.hHSC70 were provided by Y. Imamura (Matsumoto Dental University, Nagano, Japan)^21^. The pmRFP.Cx43 and pcDNA3.1.Cx43 were used previously^15^. The pEF.cyclin D1.myc and pcDNA3.1.HA-p27 were provided by P. Zou (Keio University, Tokyo, Japan)^23^. pCI-neo-FLAGhCDK4 was provided by T. Kabuta (National Institute of Neuroscience, National Center of Neurology and Psychiatry, Tokyo, Japan)^44^.

### Cell culture and transfection

HuH-7 cells were grown in DMEM containing 100 U/ml penicillin and 100 μg/ml streptomycin. These cells were plated in dishes 20 hr prior to transfection. Transient transfection with plasmids of interest was performed using Lipofectamine LTX (Invitrogen, Carlsbad, CA) according to the manufacturer’s instructions. PANC-1 cells were grown in RPMI-1640 containing 100 U/ml penicillin and 100 μg/ml streptomycin. These cells were plated in dishes 20 hr prior to transfection. Transient transfection with plasmids of interest was performed using Lipofectamine 2000 (Invitrogen) according to the manufacturer’s instructions. These transfected cells were used for the following experiments.

### RNA interference

Transient transfection of both plasmids of interest and siRNA of Hsc70 (Santa Cruz Biotechnology, CA) or Silencer negative control number 1 siRNA (NC) (Santa Cruz Biotechnology) was carried out using Lipofectamine 2000 reagent (Invitrogen) according to the manufacturer’s instructions.

### BrdU incorporation assay

For BrdU incorporation assay, HuH-7 cells and PANC-1 cells were plated onto 35-mm glass covered dishes. After 20 hr of cell seeding, the cells were transfected with various plasmids of interest. After 48 hr of transfection, the cells were labeled with 10 μM BrdU for an additional 1 hr. Then, the cells were fixed and detected by using 5-Bromo-2′-deoxyuridine Labeling and Detection Kit (Roche, Basel, Switzerland). These cells were analyzed and counted by confocal microscopy (FV1000, Olympus, Tokyo, Japan). After BrdU-positive cells were counted, the percentage of BrdU-positive cells among total counted cells was calculated.

### Immunofluorescence staining

HuH-7 cells and PANC-1 cells were plated on 35-mm glass covered dishes. After 20 hr of cell seeding, the cells were transfected with plasmids of interest. After 48 hr of transfection, the cells were fixed in 2% paraformaldehyde (PFA) in PBS for 1 hr. After fixation, the cells were permeabilized by incubation with 0.1% Triton X-100 in PBS for 10 min, blocked with 3% skim milk in PBS for 1 hr, and incubated with primary antibody in 3% skim milk solution overnight. Primary antibodies used were: anti-Cx43 antibody (Sigma, St. Louis, MO), anti-T7-tag antibody (Novagen, Madison, WI, USA), anti-cMyc-tag antibody, anti-DYKDDDDK (FLAG)-tag antibody (Wako Pure Chemical, Osaka, Japan), anti-HA-tag antibody (Y11, Santa Cruz Biotechnology), and anti-p27 antibodies (C19, Santa Cruz Biotechnology). Secondary antibodies used were: Alexa Fluor 488 goat anti-mouse IgG, Alexa Fluor 488 goat anti-rabbit IgG, and Alexa Fluor 594 goat anti-mouse IgG (Invitrogen-Molecular Probes) in 3% skim milk solution for 1.5 hr. DNA was stained with TO-PRO3 iodide (Invitrogen-Molecular Probes) for 30 min. Cells were washed carefully with PBS (3 times, 10 min each time) after incubation with primary and secondary antibodies. After immunostaining, samples were mounted and analyzed by confocal microscope (FV1000, Olympus).

### Nucleocytoplasmic localization analysis

The ratio of nuclear/cytoplasmic (N/C) fluorescence intensities was determined as previously described^15^.

### Co-immunoprecipitation assay and western blotting

Co-immunoprecipitation assay and western blotting (WB) were performed as previously described^45^. Lysates were prepared from HuH-7 cells transfected with plasmids of interest 48 hr after transfection. The antibodies used in co-immunoprecipitation were anti-Cx43 antibody (Sigma), anti-T7-tag antibody (Novagen), anti-HA-tag antibody, anti-DYKDDDDK (FLAG)-tag antibody (Wako Pure Chemical), and normal mouse and normal rabbit IgG (Santa Cruz Biotechnology). The antibodies used in WB were anti-Cx43 antibody (Sigma), anti-Hsc70 antibody (StressMarq), anti-T7-tag antibody (Novagen), anti-HA-tag antibody, anti-DYKDDDDK (FLAG)-tag antibody, anti-cMyc-tag antibody (Wako Pure Chemical), and anti-GAPDH antibody (Santa Cruz Biotechnology). The signals were measured by Image Quant LAS500 (GE Healthcare UK Ltd., Buckinghamshire, England).

### Statistical analysis

Differences between two groups were evaluated by Student’s ***t*** test except for nucleocytoplasmic localization analysis, and by Mann-Whitney U test in nucleocytoplasmic localization analysis. Differences of *p *< 0.05 were considered statistically significant.

## Additional Information

**How to cite this article**: Hino, H. *et al.* Interaction of Cx43 with Hsc70 regulates G1/S transition through CDK inhibitor p27. *Sci. Rep.* **5**, 15365; doi: 10.1038/srep15365 (2015).

## Supplementary Material

Supplementary Information
