# Supplementary material for: Interaction of Cx43 with Hsc70 regulates G1/S transition through CDK inhibitor p27
Source: Sci Rep. 2015 Oct 20;5:15365. doi: 10.1038/srep15365 (PMC4612729; doi:10.1038/srep15365)

**Interaction of Cx43 with Hsc70 regulates G1/S transition through CDK inhibitor p27**

Hitoshi Hino, Ping Dai, Tatsushi Yoshida, Tomoya Hatakeyama, Yoshinori Harada, Eigo Otsuji, Tsukasa Okuda and Tetsuro Takamatsu

**Supplemental Information**

**Supplemental Figure Legends and Figures S1-S3**

**Supplementary Figure S1**

Effects of Cx43 expression on nuclear accumulation of p27 and G1/S transition in PANC-1 cells.

(**a**) Effect of Cx43 on nuclear accumulation of p27. PANC-1 cells were transfected with control plasmid mRFP or Cx43-mRFP, and/or both Cx43-mRFP and T7-Hsc70. Immunofluorescence staining with anti-p27 antibodies was carried out. Nuclear accumulation of p27 was examined and quantified with a confocal laser scanning microscope (FV1000, Olympus, Tokyo, Japan).

(**b**) Ratio of nuclear/cytoplasmic (N/C) fluorescence intensities in p27. At least 30 transfected PANC-1 cells of control mRFP or Cx43-mRFP, and/or both Cx43-mRFP and T7-Hsc70 were examined and quantified. The data shown in (**a**) were plotted. The horizontal lines represent median values. **p* < 0.05.

(**c**) BrdU incorporation assays were carried out in PANC-1 cells. PANC-1 cells were transiently transfected with vectors encoding mRFP or Cx43-mRFP, and/or both Cx43-mRFP and T7-Hsc70, respectively. Forty-eight hours after transfection, BrdU incorporation assay was performed. The fraction of BrdU-positive cells over total number of transfected cells was determined. Data are shown as mean and SD (n = 4). **p* < 0.05.

**Supplementary Figure S2**

Full length blots.

(a) Co-immunoprecipitation of Hsc70 with Cx43. Full length blots of Figure 3a.

(b) Co-immunoprecipitation of p27 with Hsc70. Full length blots of Figure 3b.

(c) Co-immunoprecipitation of p27 with Cx43. Full length blots of Figure 3c.

(d) Co-immunoprecipitation of Cx43 and p27 with Hsc70. Full length blots of Figure 4d.

**Supplementary Figure S3**

Down-regulation of Hsc70 decreases the nuclear accumulation of the components of cyclin D1-CDK4-p27 complex.

(a) Knockdown of Hsc70 in HuH-7 cells. HuH-7 cells were transfected with mRFP and negative control siRNA (NC) or siRNA of Hsc70. After 48 hr of transfection, immunofluorescence staining with anti-Hsc70 antibody (StressMarq, Victoria, Canada) was carried out. The expression of Hsc70 was clearly knocked down by siRNA of Hsc70 in HuH-7 cells.

(b) Knockdown of Hsc70 did not affect the association of cyclin D1-CDK4-p27 complex. HuH-7 cells were transfected with equivalent plasmids of HA-p27, cMyc-cyclin D1 and FLAG-CDK4 with negative control siRNA (NC) or siRNA of Hsc70. Lysates were immunoprecipitated by control IgG or anti-FLAG-tag antibody. The immunocomplexes were analyzed on 12% SDS-PAGE followed by western blotting (WB) using anti-FLAG-tag or anti-cMyc-tag, and/or anti-HA-tag (Wako Pure Chemical) or anti-Hsc70 antibodies (B6, Santa Cruz Biotechnology) . Cropped blots are shown.

(c, d and e) Subcellular localization of CDK4 (c), cyclin D1 (d) and p27 (e) in Hsc70-knocked down and control NC HuH-7 cells. HuH-7 cells were transfected with equivalent plasmids of HA-p27, cMyc-cyclin D1 and FLAG-CDK4 and with NC or siRNA of Hsc70. After 48 hr of transfection, immunofluorescence staining with anti-FLAG-tag or anti-cMyc-tag, and/or anti-HA-tag antibodies was carried out, respectively. The nuclei were stained with TO-PRO3.


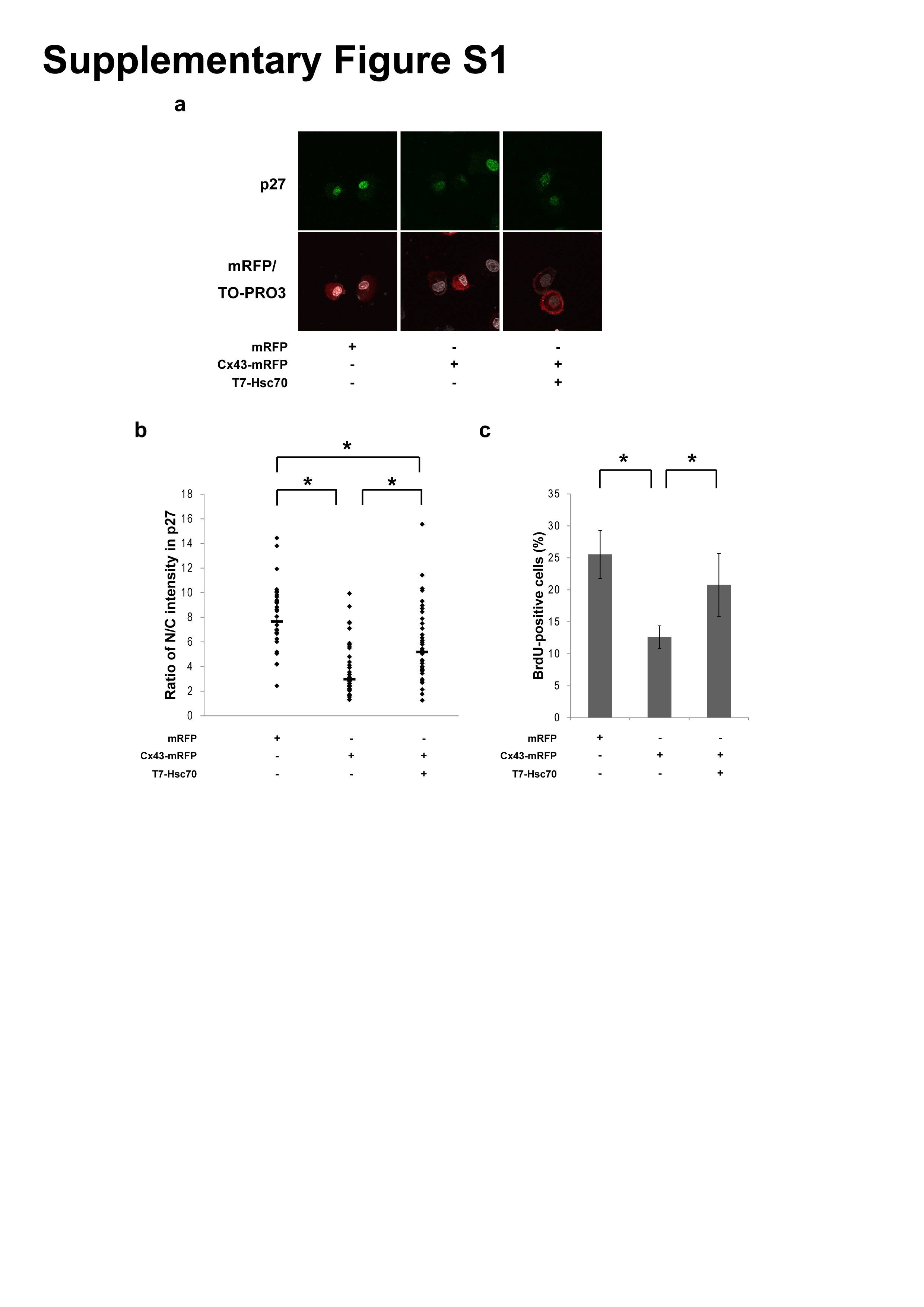


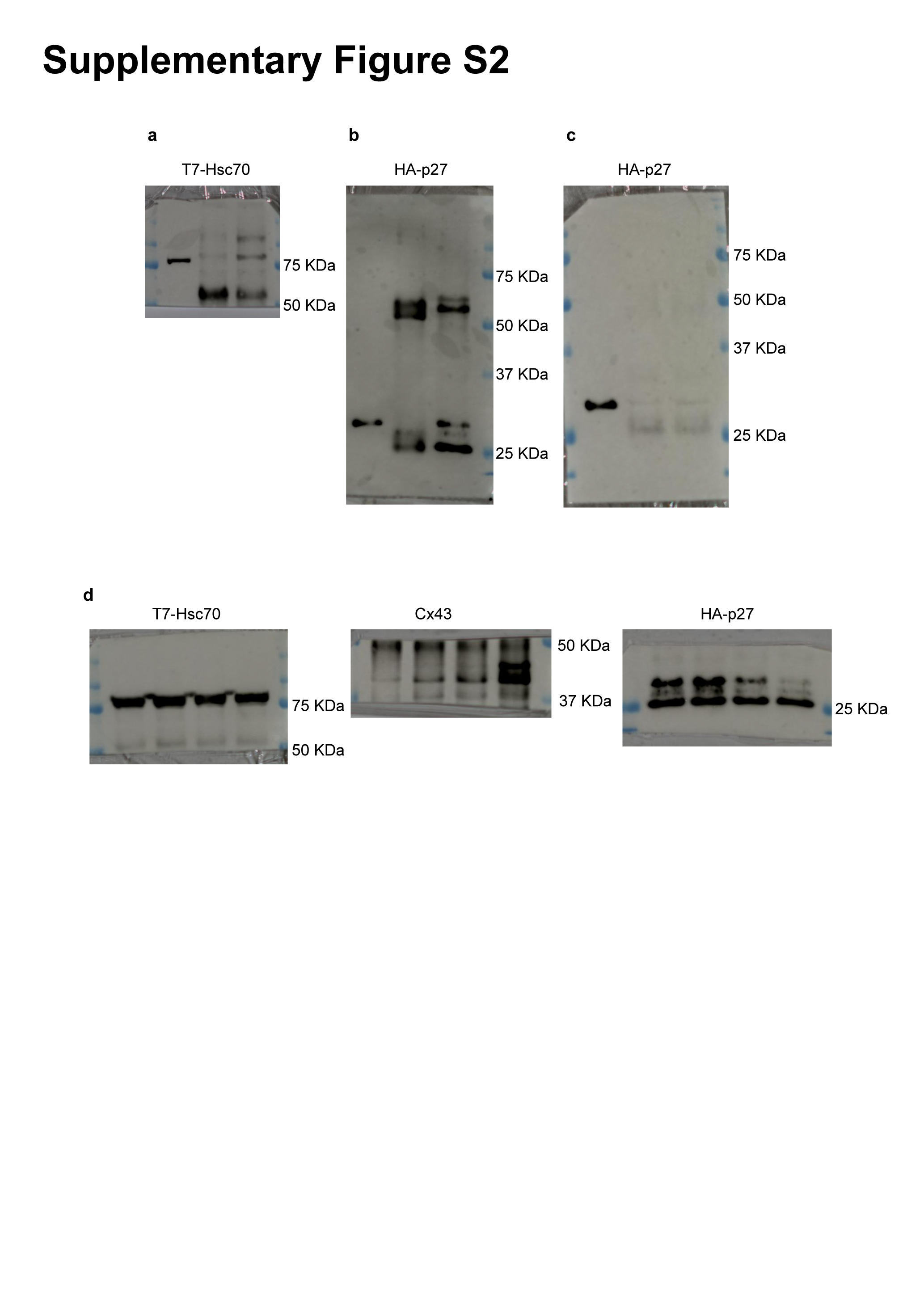


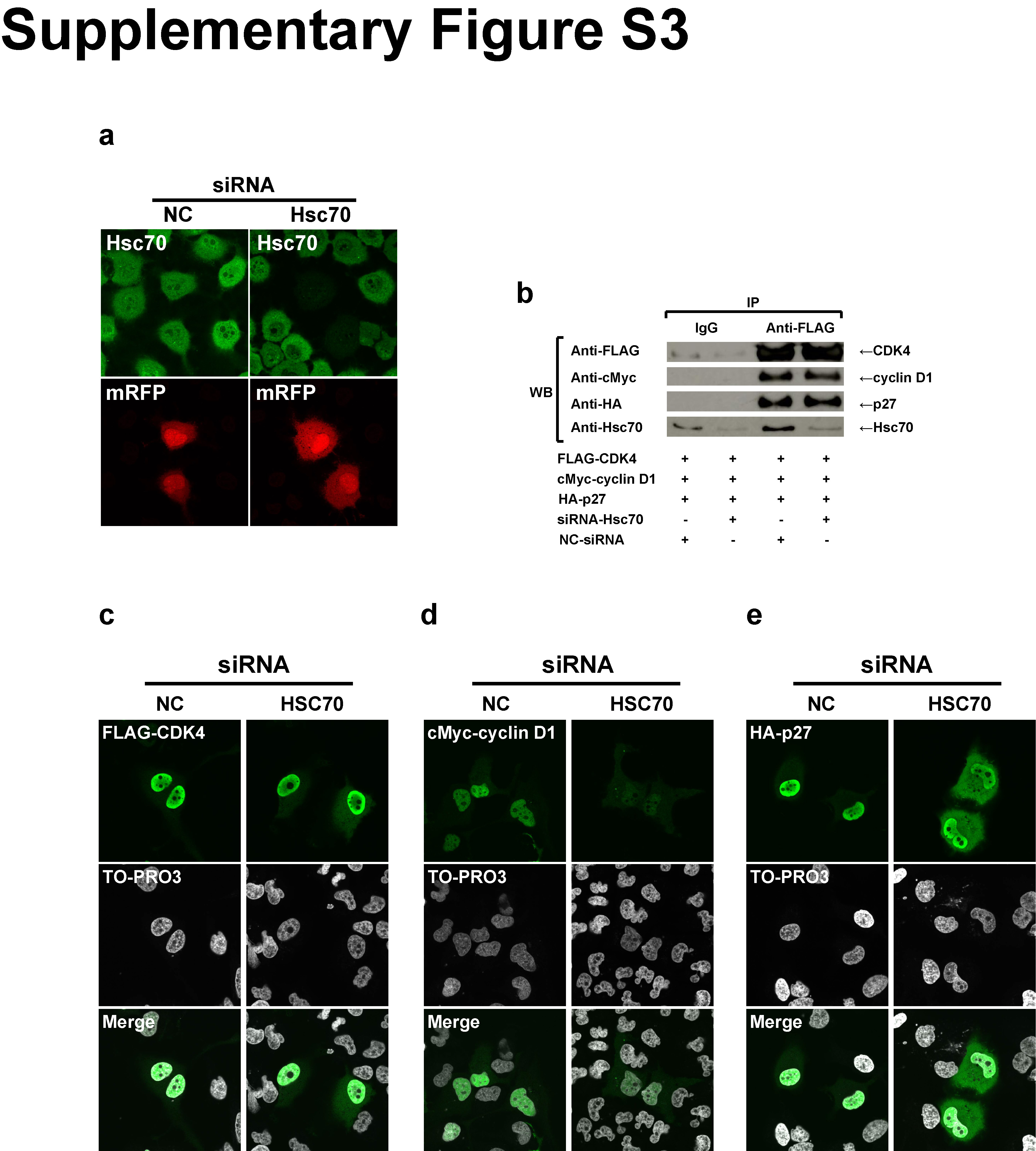

Supplement: Supplementary Information [file srep15365-s1.doc]
